# A Fast and Reliable Method Based on QCM-D Instrumentation for the Screening of Nanoparticle/Blood Protein Interactions

**DOI:** 10.3390/bios13060607

**Published:** 2023-06-02

**Authors:** Mariacristina Gagliardi, Laura Colagiorgio, Marco Cecchini

**Affiliations:** NEST, Istituto Nanoscienze-CNR and Scuola Normale Superiore, Piazza San Silvestro, 56127 Pisa, Italy; laura.colagiorgio@nano.cnr.it (L.C.); marco.cecchini@nano.cnr.it (M.C.)

**Keywords:** QCM-D, nanoparticles, blood proteins, protein corona, hemocompatibility

## Abstract

The interactions that nanoparticles have with blood proteins are crucial for their fate in vivo. Such interactions result in the formation of the protein corona around the nanoparticles, and studying them aids in nanoparticle optimization. Quartz crystal microbalance with dissipation monitoring (QCM-D) can be used for this study. The present work proposes a QCM-D method to study the interactions on polymeric nanoparticles with three different human blood proteins (albumin, fibrinogen and γ-globulin) by monitoring the frequency shifts of sensors immobilizing the selected proteins. Bare PEGylated and surfactant-coated poly-(D,L-lactide-*co*-glycolide) nanoparticles are tested. The QCM-D data are validated with DLS and UV-Vis experiments in which changes in the size and optical density of nanoparticle/protein blends are monitored. We find that the bare nanoparticles have a high affinity towards fibrinogen and γ-globulin, with measured frequency shifts around −210 Hz and −50 Hz, respectively. PEGylation greatly reduces these interactions (frequency shifts around −5 Hz and −10 Hz for fibrinogen and γ-globulin, respectively), while the surfactant appears to increase them (around −240 Hz and −100 Hz and −30 Hz for albumin). The QCM-D data are confirmed by the increase in the nanoparticle size over time (up to 3300% in surfactant-coated nanoparticles), measured by DLS in protein-incubated samples, and by the trends of the optical densities, measured by UV-Vis. The results indicate that the proposed approach is valid for studying the interactions between nanoparticles and blood proteins, and the study paves the way for a more comprehensive analysis of the whole protein corona.

## 1. Introduction

Acoustic sensors are devices composed of piezoelectric materials, combining mechanical waves and electrical signals to provide detection [1]. Thanks to their versatility and sensitivity, this class of sensors has become increasingly important in various fields and, in particular, in biomedical applications [2,3]. Bio-related applications of acoustic sensors mostly concern the analysis of various biomolecules and cells, but they have shown great potential also in the early detection of diseases and drug screening. For such applications, two types of acoustic sensors, namely surface acoustic wave (SAW) [4,5,6] and bulk acoustic wave (BAW) [7,8] are widely investigated.

In addition to cited detections, acoustic sensors have also been used to study specific characteristics of nanoparticles used in nanomedicine [9,10,11]. Polymers are commonly used in nanoparticle preparation, and their interaction with the host environment is crucial for their safety. Drug-loaded nanoparticles are largely explored as drug nanocarriers, in particular for the administration of toxic drugs to limit side effects and provide targeted drug release [12]. Several nanovectors are under investigation for systemic administration via intravenous injection. In such cases, deep knowledge of the interactions between nanoparticles and blood proteins is fundamental. The so-called protein corona is a crucial factor in the interactions between nanoparticles and biological systems [13]. The protein corona is a coating that spontaneously forms around nanoparticles when exposed to a biological medium. Such a coating consists of proteins, lipids, and other biomolecules from the surrounding environment [14]. The protein corona can alter the size, zeta potential, morphology, and aggregation state of the nanoparticles [15] and, consequently, their interactions with the physiological system, inducing adverse reactions and accelerated clearance [16]. The study of the protein corona through conventional analytical techniques is complex and requires great expertise [17]. However, a fast and reliable screening of the interactions between nanoparticles and serum, plasma, and blood proteins can be achieved by acoustic biosensors. In a biosensor, the bioreceptor or probe is the molecule used to functionalize the surface of the sensitive element. The probe is selected to bind the analyte in a specific manner. Thus, proper selection of the probe is fundamental to increase the specificity of the detection. Regarding the formation of a protein corona around polymer nanoparticles, useful molecules that could be used in a biosensor are the blood proteins involved in this process. The functionalization of a biosensor with blood proteins, combined with the analysis of nanoparticle dispersion, can work to measure the intensity of the interactions between the immobilized probe and the nanoparticle sample. In an acoustic sensor, these interactions are given by the number of nanoparticles retained by the probe layer.

A few studies have characterized the protein corona formation on polymeric nanoparticles using the quartz crystal microbalance with dissipation monitoring (QCM-D). The QCM-D apparatus allows for the simultaneous monitoring of the variation of the vibrational frequency of the sensor (frequency shift) and the variation of the energy dissipation (dissipation shift) when a molecule interacts with the sensor surface. The frequency shift can be directly related to the amounts of molecules interacting with the sensor surface, according to the Sauerbrey equation (Equation (1)):

(1) $$∆m=-C\times \frac{∆f_{n}}{n}$$

In this equation, Δ*m* is the areal mass, or the mass variation over the sensor surface (ng cm^−2^), Δ*f_n_* is the frequency shift (Hz) measured for the selected harmonic *n*, *n* is the number of the odd harmonic, and *C* is the sensitivity constant. *C* is equal to 17.7 ng cm^−2^ Hz^−1^ for a crystal with a fundamental resonance of 5 MHz.

With this technique, the interaction between nanoparticles and biological molecules, by varying the nanoparticle size, surface charge, and functionalization [18], was studied. A very extensive state-of-the-art technique concerning the analysis of the interaction between nanoparticles and proteins in an aqueous environment has been proposed by Xu and collaborators [19]. This work exhaustively illustrates all the possible interaction models and how to analyze the experimental data obtained to have a better understanding of the measured phenomena. Concerning the interactions between nanoparticles and blood components, an interesting study reports the QCM-D characterization of the interactions between a lipid bilayer and carboxylated polystyrene nanoparticles coated with different kinds of protein corona [20]. The study found that the ‘soft’ protein corona, composed of loosely bounded proteins, induced a permanent alteration in the lipid bilayer structure, while the ‘hard’ protein corona, composed of strongly bounded proteins, weakly interacted with a lipid bilayer. However, QCM-D is a very versatile technique with which the interactions of nanoparticles have also been studied with other blood components, such as platelets [21]

The study of the protein corona is crucial for the development of safe and effective nanoparticles. The QCM-D technique can provide reliable screening of interactions with protein corona-related blood proteins and has been used in several studies to characterize protein corona formation on polymeric nanoparticles. However, the results obtained from QCM-D measurements should be interpreted in the whole context of the specific system under investigation, and other techniques should be used in conjunction with QCM-D to provide a comprehensive characterization of the protein corona.

In the present work, the QCM-D technique is applied to a rough evaluation of the protein corona formation around poly(D,L-lactide-*co*-glycolide) nanoparticles. The QCM-D sensors were functionalized with three different blood proteins, namely albumin from human serum (HSA), fibrinogen type I from human plasma (HPF), and γ-globulins from human blood (HBG), and the adhesion of the nanoparticles to the functionalization layers was evaluated. Three nanoparticles with different protein affinities were tested, and the system was validated with dynamic light scattering (DLS) and UV-Vis experiments. This research provides a valid method to characterize the interactions between tested nanoparticles and three blood proteins commonly abundant in the protein corona. The work also reports a size analysis of nanoparticles incubated with blood proteins using DLS, and the variation in the optical density of nanoparticle/blood protein solutions with time using UV-Vis spectroscopy.

## 2. Materials and Methods

All the reagents used for this work were purchased from Sigma Aldrichif not otherwise stated.

For the nanoparticle preparation, Resomer RG503H (PLGA, Mw 24–38 kDa, carboxyl-terminated) and methoxy polyethylenglycol-*block*-poly(D,L-lactide-*co*-glycolide) (mPEG-b-PLGA, PEG Mw 5 kDa, PLGA Mw 20 kDa, hydroxyl-terminated) were used as polymers, sodium cholate was used as the surfactant, and absolute ethanol, water, and acetone were used as solvents. Human serum albumin (HSA, Mw 66.5 kDa), human plasma fibrinogen (HPF, dimer, Mw 340 kDa), and human blood γ-globulin (HBG, Mw 150 kDa) were used as the proteins for the nanoparticle testing. Phosphate buffer solution (PBS, 140 mM NaCl, 10 mM phosphate buffer, and 3 mM KCl, pH 7.4 at 25 °C) was used as the medium for nanoparticle testing. In the QCM-D experiments, 12-mercaptododecanoic acid (12-MCA, Mw 232.4 Da, purity degree 96%) and 1,4-dithiothreitol (DTT) were used to form the adlayer, and N-(3-Dimethylaminopropyl)-N′-ethylcarbodiimide hydrochloride (EDCl) and N-hydroxysuccinimide (NHS) were used as coupling agents.

### 2.1. Nanoparticle Preparation and Characterization

The nanoparticles were prepared by solvent displacement using a polymer solution with a fixed concentration and a precipitation medium. We used two different precipitation media: the first was composed of a water/ethanol mixture (1/1 *v*/*v*), and the second was composed of a 2% sodium cholate solution in water. A solution of the selected polymer in acetone (10 mL, 5 mg/mL) was added dropwise to the precipitation medium (50 mL) and vigorously stirred for 4 h. At the end of the procedure, the solution was collected in a plastic vial and stored at 4 °C before use.

The size and ζ-potential of the freshly prepared nanoparticles were measured by DLS (Malvern Panalytica Z-Zetasizer, Malvern, UK). Samples for size measurements were obtained by diluting 5 µL of the nanoparticle stock solution in 200 µL of water. After the size measurement, the same samples were further diluted with water to 800 µL and the ζ-potential was measured. The measurements were performed in triplicate.

### 2.2. QCM-D Measurements

The QCM-D (E4 model, Q-Sense AB, Sweden) measurements were performed with polished AT-cut quartz crystals (gold electrodes, fundamental resonance frequency f_0_ = 5 MHz, overall diameter = 14 mm, gold sensor diameter = 10 mm, quartz thickness = 300 µm, Biolin Scientific, Västra Frölunda, Sweden) in static mode (stop flow), with fluidic cells thermostatted at 37 °C. The apparatus records the resonance frequency shift (ΔF) and energy dissipation (ΔD) simultaneously for up to 13 overtones by exciting the fundamental resonance frequency of the crystal. In this work, we monitored the 3rd, 5th, 7th, 9th, and 11th resonances and calculated the ΔF and ΔD as the difference between the baseline (water) and the signals obtained after rinsing with water. We also checked if the ΔD values were suitable for the application of the Sauerbrey model (Equation (1)). One of the proposed criteria to check is to have ΔD < 2.0 × 10^−6^ [22]. In our case, the ΔD values were higher but close to the limiting value. Thus, we considered the Sauerbrey equation valid for all the functionalizations.

#### 2.2.1. Sensor Functionalization

The QCM-D sensors were functionalized with the blood proteins as probes to test the affinity of the nanoparticles toward the selected protein. Before use, the quartz crystals were rinsed with 2% sodium dodecyl sulphate and then dried and treated with plasma oxygen (Femto Diener) for 2 min at a power of 100 W, immersed in a 5:1:1 solution of water, ammonia (32% *v*/*v*), and oxygen peroxide (25% *v*/*v*) at 75 °C for 15 min, rinsed with water and after with isopropanol, and treated with plasma oxygen again (2 min, 100 W).

**Figure 1:**
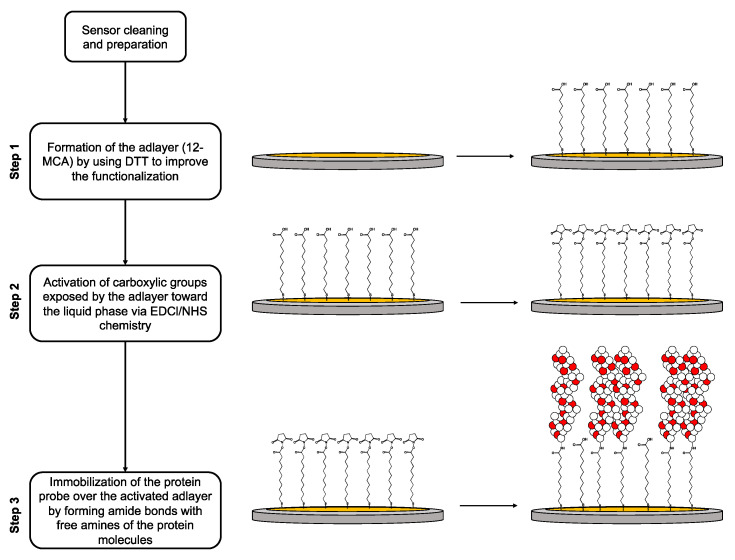
Schematization of QCM-D sensor functionalization (not in scale): in Step 1, the bare Au electrode is functionalized with a 12-MCA solution containing DTT as reducing agent, to provide the adlayer with carboxylic acid functionalities exposed over the surface; in Step 2, the carboxylic acid groups are activated via EDCl/NHS chemistry to provide N-hydroxy succinimide esters; in Step 3, the activated adlayer reacts with the selected blood protein to provide the probe layer.

The gold surface of the crystal quartz can be easily modified by forming the functional layer via thiol-gold chemistry. Since the selected proteins did not contain functional groups useful for the thiol-gold chemistry, we preliminarily prepared an adlayer composed of 12-MCA. To do this, the sensors mounted in the microfluidic chambers were previously pre-rinsed with a mixture of water/ethanol (1/1 *v*/*v*), and the data were acquired for 2 min. Then, a solution of 12-MCA (2 mg/mL) in a mixture of water/ethanol (1/1 *v*/*v*) containing DTT (reducing agent) 1× mol/mol of free thiols was injected into the microfluidic chamber (10 min). Finally, the sensors were rinsed with water/ethanol (2 min) and pure water (2 min). The obtained 12-MCA adlayer had the carboxylic functionalities exposed toward the water phase and available for the following protein conjugation. To obtain the conjugation, the carboxylic functionalities were activated by injecting into the QCM-D chambers a water solution containing EDCl/NHS (20 mM each, 10 min), and then the sensors were rinsed with water (2 min). The last step was the conjugation of the probe. The sensors were previously rinsed with PBS (2 min), and then a solution of the selected protein (1 mg/mL) in PBS was injected (30 min). Finally, the sensors were rinsed with PBS (2 min) and water (2 min). A schematization of the functionalization process is shown in Figure 1.

#### 2.2.2. Sample Detection

After the functionalization, a sample solution containing 500 µg/mL in PBS was prepared. The sensors were pre-rinsed with PBS (2 min), and then the sample solution was injected (30 min). Finally, the sensors were rinsed with buffer (2 min) and water (2 min).

### 2.3. In Cuvette Characterization

The interactions between the nanoparticles and blood proteins were also measured *in cuvette* experiments. We measured i. the variation of the nanoparticle size during incubation with blood proteins and ii. the optical density changes with time in the presence of the selected proteins.

For the first experiment, 80 µL of the nanoparticle stock solution, corresponding to 0.1 mg of nanoparticles, was added to 320 µL of the solution of the selected protein in PBS. The final concentration of the proteins was 40 mg/mL for HSA, 3 mg/mL for HPF, and 12 mg/mL for HBG. Such concentrations were selected to fall within the physiological human range (35–44 g/L for HSA [23], 2–4 g/L for HPF [24], 6–20 g/L for HBG [25]). The prepared samples were immediately added to a quartz cuvette, thermostatted at 37 °C, and measured by DLS. The nanoparticle size was acquired every 5 min up to 60 min. Samples only containing nanoparticles dispersed in PBS, and the blank protein solutions were measured as controls. An estimation of the nanoparticle concentrations in the incubated samples was monitored via DLS measurements by monitoring the mean photon count rate. This estimation was possible because the mean photon count rate is proportional to the nanoparticle number concentration [26]. The mean photon count rate was acquired with a fixed attenuator.

In the second experiment, the samples were prepared with the same procedure followed for the DLS measurements, but the samples were stored in Eppendorf tubes and thermostatted with a thermomixer at 37 °C. After 30 min and 60 min, the samples were added to a quartz cuvette, and the UV-Vis spectrum was acquired in the range from 350 to 220 nm (JASCO V550 spectrophotometer, JASCO Europe, Cremello, Italy). The optical density at 280 nm was measured to qualitatively monitor the sample turbidity, which is correlated to the formation of large aggregates, precipitation, and flocculation. The samples only containing nanoparticles dispersed in PBS and the blank protein solutions were measured as controls. The tests were performed in triplicate.

### 2.4. Data Analysis and Statistics

The DLS raw data were collected from the instrument and fitted with a beta distribution function to calculate the mode (peak value), the polydispersion index (PDI), and the full width half maximum (FWHM). For the measurements of the freshly prepared samples, the data are reported as the mean of the mode value ± SD, the measurements were performed in triplicate, and the size distributions by intensity were considered. For the ζ-potential analysis, the data are reported as the mean value ± SD, and the measurements were performed in triplicate. For the measurements of the samples incubated with the blood proteins, the data are reported as the mode value ± FWHM, and the size distributions by intensity were considered.

The QCM-D raw data were collected from the instrument, and a drift correction was performed when needed using an in-house script running on the commercial software MATLAB (MathWorks, Natick, MA, USA). The values of the shifts after rinsing were calculated as the difference between the value of ΔF or ΔD measured at the end of the analysis (after rinsing with water) and the baseline (water). The analysis of the transient sample/probe interactions was obtained considering the data collected after the sample injection and before the rinsing. Both the ΔF and ΔD data vectors were treated to have a value of zero as the first point. The ΔF and ΔD are reported as the mean value ± SE, the measurements were performed in quadruplicate, the Δm was calculated from the ΔF using the Sauerbrey equation (Equation (1)), and the results are reported as the mean value ± SE. In the boxplots, the values are indicated as outliers if they are greater than Q_3_ + w × (Q_3_–Q_1_) or less than Q_1_–w × (Q_3_–Q_1_), where w is the multiplier ± 2.7 σ and Q_1_ and Q_3_ are the 25th and 75th percentiles of the sample data, respectively. The number of molecules immobilized over the sensor surface was calculated starting from the values of ΔF_5_ after rinsing with water. Such values entered the Sauerbrey equation, and then the calculated values of the areal mass Δm_5_ (ng cm^−2^) were divided by the molecular weight of the protein (ng nmol^−1^), multiplied by 10^−9^ to convert the data in mol cm^−2^, and, finally, multiplied by the Avogadro constant N_A_ (1.022 × 10^23^ molecules mol^−1^).

For the UV-Vis measurements, the acquired spectrum data were imported into the commercial software Excel (Microsoft Corporation, Washington, DC, USA). The reported data are the optical densities measured at 280 nm. The optical densities are reported as the mean ± SE, the OD percent variations are calculated as the ratio between the OD change with respect to the starting time and the OD value at the starting time, and the measurements were performed in triplicate.

## 3. Results

### 3.1. Nanoparticle Characterization

**Figure 2:**
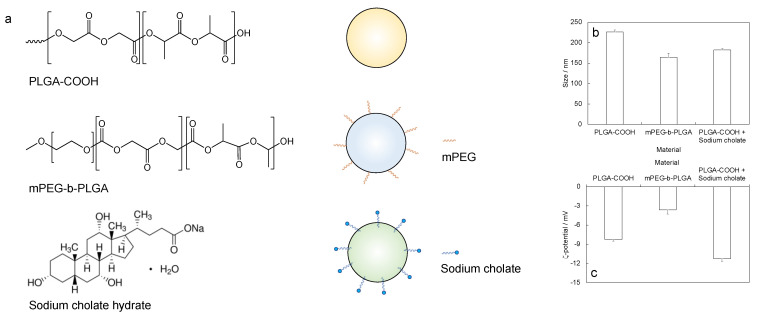
Nanoparticle preparation: (**a**) chemical formulas of reagents (poly(D,L-lactide-*co*-glycolide carboxyl-terminated, PLGA-COOH, methoxy poly(ethylenglycol)-*block*-poly(D,L-lactide-*co*-glycolide, MPEG-b-PLGA, sodium cholate hydrate as surfactant) and corresponding schematization of the formed nanoparticles; (**b**) size and (**c**) surface ζ-potential, measured in samples containing freshly prepared nanoparticles.

The freshly prepared nanoparticles were supposed to have different surface properties (a schematization is reported in Figure 2a). Thus, the size (Figure 2b) and ζ-potential (Figure 2c) were expected to differ between the different formulations. The larger size was measured for the PLGA-COOH nanoparticles obtained in the water/ethanol medium (peak value: 226 ± 5 nm, PDI: 0.42 ± 0.07, FWHM: 100 ± 7 nm) and the lower size was measured for the mPEG-b-PLGA nanoparticles obtained in the water/ethanol medium (peak value: 164 ± 10 nm, PDI: 0.63 ± 0.12, FWHM: 85 ± 12 nm), while a middle size was measured for the PLGA-COOH nanoparticles obtained in the medium containing sodium cholate (peak value: 182 ± 3 nm, PDI: 0.64 ± 0.03, FWHM: 98 ± 1 nm). The surface ζ-potential values were negative in all the cases, ranging from −3.6 ± 0.7 mV to −11.2 ± 0.4 mV, and were comparable among the selected samples.

### 3.2. QCM-D Measurements

**Figure 3:**
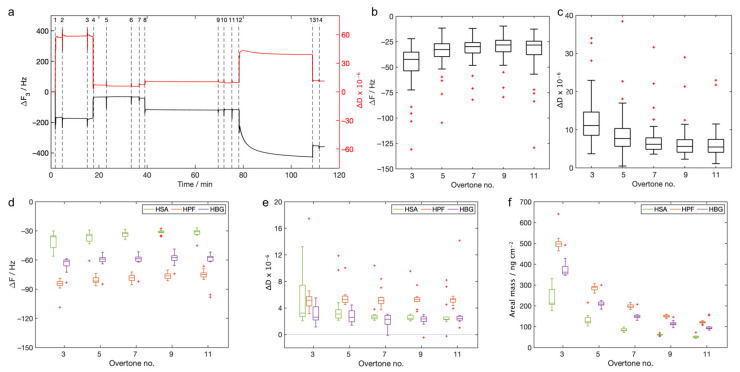
QCM-D analysis: (**a**) traces of ΔF (black) and ΔD (red) registered in the experiments (only one plot is reported as a representative example. In this example, the probe was HPF, and the analyzed sample was PLGA-COOH + sodium cholate), the third overtone is reported (events: 1 pre-rinsing with water/ethanol, 2 injection of the adlayer solution, 3 rinsing with water/ethanol, 4 rinsing with water, 5 injection of the activation solution, 6 rinsing with water, 7 pre-rinsing with PBS, 8 injection of the probe solution, 9 rinsing with PBS, 10 rinsing with water, 11 pre-rinsing with PBS, 12 injection of the sample, 13 rinsing with PBS, 14 rinsing with water); (**b**) ΔF and (**c**) ΔD measured for the adlayer formation after rinsing (baseline water, n = 40); (**d**) ΔF and (**e**) ΔD measured for the probes after rinsing (baseline water, n = 14 for HSA, n = 12 for HPF and HBG); (**f**) areal masses of the probes, calculated with the Sauerbrey equation (Equation (1)) for all the overtones considered. In all the reported plots, red crosses are the outliers.

The whole traces (Figure 3a) of ΔF were registered and indicated a decrease after each event with respect to the baseline, while the traces of ΔD highlighted an increase.

The final values of ΔF measured for the adlayer formation (Figure 3b), calculated as the difference between the frequency value measured after rinsing and the baseline, ranged from −30 Hz to −55 Hz for all the considered overtones. The values of ΔD were less than 12 × 10^−6^ for all the overtones considered (Figure 3c). The final values of ΔF and ΔD measured for the probes were also not significantly dependent on the overtone number. The final ΔFs measured after the rinsing were larger for the HPF, smaller for the HSA, and median for the HBG (Figure 3d). The values of ΔD were in the range of 3 × 10^−6^ to 5 × 10^−6^ (Figure 3e). These values were reasonably closer to the limiting value for the application of the Sauerbrey equation, and this allowed us to calculate the areal mass using this model. The areal masses were strongly dependent on the overtone and ranged from 50 ng cm^−2^ to 500 ng cm^−2^ (Figure 3f).

**Figure 4:**
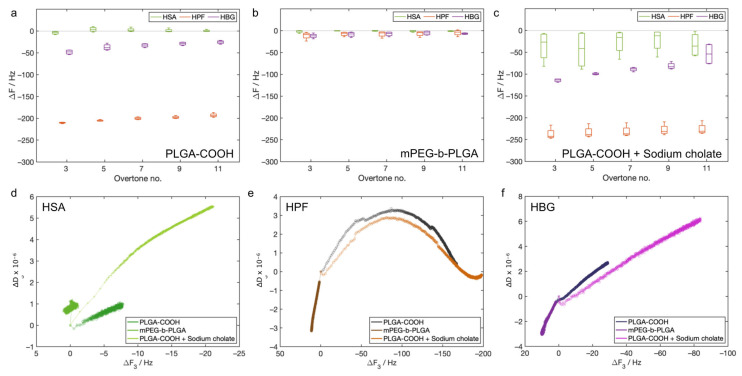
Sample detection and analysis of the transient sample/probe interactions: (**a**–**c**) ΔF shifts after rinsing, measured for samples PLGA-COOH, mPEG-b-PLGA, and PLGA-COOH + sodium cholate, respectively (n = 4 for each sample); (**d**–**f**) ΔD_3_ vs. ΔF_3_ measured during the detection, values are reported as the mean of 4 sensors, pre-rinsing and rinsing phases were excluded.

The sample detections (Figure 4a–c) indicated that the HSA probe had a poor interaction with all the analyzed samples. A larger ΔF was measured only for the sample PLGA-COOH + sodium cholate with this protein. The signals measured with the HPF probe were very large for both PLGA-COOH-based samples, in particular in the sample containing the surfactant. On the other hand, the mPEG-b-PLGA sample gave a very low signal (in the range of −3 Hz to −8 Hz). The HBG probe provided intermediate values with respect to the HSA and HPF. Such signals were significant for PLGA-COOH and PLGA-COOH + sodium cholate, while the ΔF was low for mPEG-b-PLGA (in the range of −9 Hz to −12 Hz).

We also considered the transient sample/probe interactions (Figure 4d–f). The analysis was performed considering the QCM-D data gathered after the sample injection and before rinsing. This analysis showed that the transient phase was different for almost every sample/probe pair. The measured frequency range for the HSA was up to −20 Hz, while larger values were measured for the HPF (up to −200 Hz) and HBG (up to −85 Hz). The values of ΔD were small in all the cases (between −4 × 10^−6^ and 8 × 10^−6^).

### 3.3. DLS Measurements

**Figure 5:**
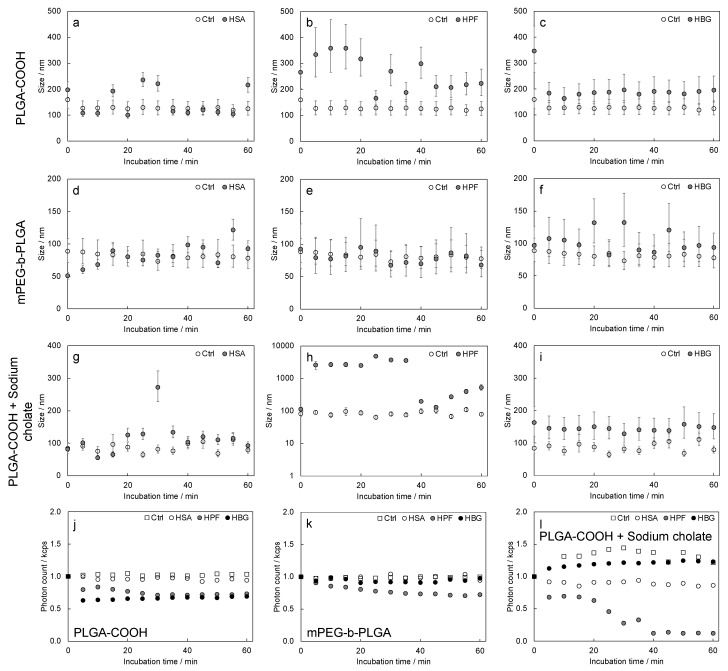
DLS analysis: mean size values measured vs. time for (**a**–**c**) PLGA-COOH; (**d**–**f**) mPEG-b-PLGA; and (**g**–**I**) PLGA-COOH + sodium cholate, respectively; (**j**–**l**) photon count rates.

The size of the nanoparticles was monitored during incubation in the blood protein to evaluate any variation occurring after the nanoparticle/protein interaction (Figure 5). The results indicated that no detectable size increases occurred in the samples incubated with HSA, only small increases were measured in the samples incubated with HBG, and unusual behavior was detected with HPF. For the last protein, we did not detect any size changes for the sample composed of mPEG-b-PLGA, while very strong size increases were detected for the PLGA-COOH and PLGA-COOH + sodium cholate samples at early times.

The mean photon count rate was almost constant with time in all the cases for the mPEG-b-PLGA samples, while it decreased for the PLGA-COOH nanoparticles incubated with HPF and HBG, and a very large decrease was detected for the sample PLGA-COOH + sodium cholate/HPF.

### 3.4. UV-Vis Measurements

**Figure 6:**
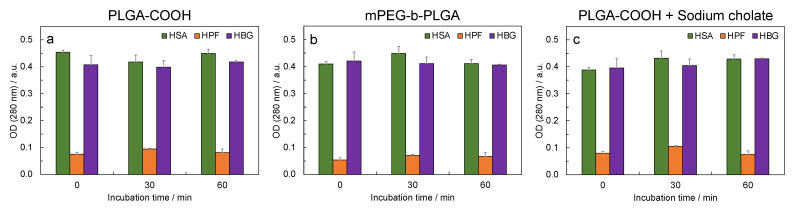
UV-Vis analysis: optical densities measured once resuspended, and after 30 min and 60 min of incubation in the selected blood protein: (**a**) PLGA-COOH, (**b**) mPEG-b-PLGA, and (**c**) PLGA-COOH + sodium cholate.

The optical density (Figure 6) of the samples incubated with the protein was monitored at three fixed times: once resuspended, after 30 min, and after 60 min. The nanoparticles resuspended in PBS exhibited a signal at 280 nm related to scattering phenomena. However, this signal appeared not to be relevant in the detection of the optical densities of the samples incubated with blood proteins. The samples resuspended in HSA and HBG did not show significant differences, except for the PLGA-COOH + sodium cholate sample, for which the optical density increased with time. A similar trend was detected for fibrinogen, except for the PLGA-COOH + sodium cholate sample. In this case, at the later time, we detected a visible flocculation. Thus, the measurement may be less accurate than the others.

## 4. Discussion

The proposed study indicated that QCM-D can be successfully used to analyze the interactions between polymer nanoparticles and blood proteins. The study implied the functionalization of the sensors with three selected blood proteins: human serum albumin, human plasma fibrinogen, and human blood γ-globulin. Such proteins were selected because they are the most abundant in human blood [27] and were reported to be involved in the formation of the protein corona after the systemic administration of nanoparticles [28]. Our study indicated a reliable procedure for the functionalization of the QCM-D sensors based on the use of a proper adlayer composed of 12-mercaptododecanoic acid, which enabled the immobilization of the proteins over the sensor surface via EDCl/NHS chemistry. The frequency shifts measured after the sensor functionalizations were, in all cases, negative. Negative values of the frequency shifts are typically associated with mass loading. We checked the values of the energy dissipation shifts for the functionalizations. The results, in all cases, were reasonably low to apply the Sauerbrey equation for the calculation of the areal mass or the mass of the probe immobilized per unit of the sensor surface. The values of the areal mass were lower for the HSA, higher for the HPF, and median for the HBG. This was in line with our expectations, considering that protein molecular weights have the same trends. Despite the different areal masses, the molecular coverage of the sensor surface was similar for all the probes. This was confirmed by the estimation of the number of molecules per unit or surface area, resulting in around 1 × 10^11^ molecules cm^−2^ (HSA), 5 × 10^10^ molecules cm^−2^ (HPF), and 9 × 10^10^ molecules cm^−2^ (HBG), which were calculated from the data gathered from the 5th overtone. However, we found a dependence of the areal mass on the frequency. This is an indication of a certain degree of viscoelasticity of the layer. Further investigation would be interesting for better comprehension of the probe behavior, but we do not expect different interactions with the nanoparticles.

Signals related to sample detection, stemming from the number of nanoparticles retained by the functionalization layer over the sensor surface, indicated that the HSA probe did not strongly interact with the selected materials PLGA-COOH and mPEG-b-PLGA. The values of ΔF were larger for the sample obtained in the presence of the surfactant sodium cholate. In this case, we can hypothesize that the electrical cationic charge of the surfactant, used to improve the solubilization of hydrophobic polymer nanoparticles in an aqueous medium, interacted with the protein. The HPF probe was the most involved in the sample/probe interactions. This protein provided very large ΔF during interaction with nanoparticles composed of PLGA-COOH, obtained both with and without the surfactant. Such behavior indicated that the surface of the tested nanoparticles had a strong affinity toward the protein, in particular when covered with surfactant molecules. On the other hand, the mPEG-b-PLGA nanoparticles did not show significant interactions with the HPF. Similar behavior was expected, and it is in line with the literature reporting good stealth properties for PEGylated nanoparticles [29]. The HBG probe showed intermediate behavior between the HSA and HPF, and the ΔF values registered high for the PLGA-COOH and PLGA-COOH + sodium cholate nanoparticles and was negligible for the mPEG-b-PLGA nanoparticles. From this analysis, we can conclude that PEGylation improves the compatibility of nanoparticles with blood proteins with respect to the bare PLGA copolymer, while the surfactant seems to make this aspect worse.

For all the nanoparticles analyzed, the surface charges were quite similar (in the range of −3.6 mV to −11.2 mV). Thus, we can exclude that the different behavior is mainly related only to differences in electrostatic interactions with the proteins.

The trends of the slope of the curve ΔD vs. ΔF, recorded during the sample detections, indicated different behaviors for each nanoparticle/probe pair. Starting from the HSA, the slope obtained from the detection of the PLGA-COOH nanoparticles was very low compared to that obtained with the PLGA-COOH + sodium cholate nanoparticles in a comparable ΔF range. It indicated that the probe had a stiffer mechanical behavior with the PLGA-COOH nanoparticles than with the PLGA-COOH + sodium cholate nanoparticles. In the detection of the mPEG-b-PLGA nanoparticles, the slope was comparable to that obtained with the PLGA-COOH nanoparticles. The trend of the curve obtained in the PLGA-COOH + sodium cholate nanoparticle detection was not linear in all the range, indicating a conformational change in the probe layer over time. The conformational change in the HSA induced by the PLGA-COOH + sodium cholate nanoparticles was highlighted by a slope decrease in the higher ΔF range. Such behavior can be attributed to increased stiffness of the probe layer and a higher packing density [1,30]. A conformational change during the sample detection was mostly marked in the HPF with the PLGA-COOH samples, both with and without the surfactant. In these measurements, the slope of the curve had a maximum and then changed the sign, from a positive slope value to a negative one. This happened at a ΔF around −80 Hz, registered in the first minutes after the sample injection. This marked behavior can be attributed to a mass deposition (ΔF < 0) and a progressive stiffening of the functionalization layer (ΔD > 0 but decreasing). The nanoparticles composed of mPEG-b-PLGA led to ΔF > 0 and ΔD < 0. Similar behavior is generally attributed to a mass loss, but, in our case, there was no evidence of mass loss of the functionalization layer. However, we can suppose that the amphiphilic mPEG-b-PLGA molecule acted as a surfactant, disrupting the probe layer packing but without mass losses. The antifouling effect due to PEGylation is commonly attributed to the hydrophilicity of PEG and the “water barrier” generated on the PEGylated surface. The tightly bound water layer is a physical and energetic barrier, which prevents protein adsorption on the surface. It was reported that, for such materials, the interactions of PEGylated surfaces with proteins can be facilitated if water molecules are expelled from both nanoparticles and proteins [31]. In our work, we can hypothesize that some weak and labile nanoparticle/probe interactions occurred in the transient phase. Due to such interactions, part of the hydration water was expelled from the functionalization layer, causing a slight ΔF increase. The detections with the HBG did not show conformational changes after interaction with the nanoparticles. We can only highlight that the PLGA-COOH nanoparticles made the probe layer less stiff than the PLGA-COOH + sodium cholate nanoparticles. The samples composed of mPEG-b-PLGA had a similar effect detected for the HPF.

The QCM-D data were perfectly in line with the size measurements. We detected by DLS that the size of all the samples did not increase upon incubation with the HSA, except for the sample PLGA-COOH + sodium cholate, for which we detected an average size increase of 38%, calculated with respect to the size measured upon dispersion in the protein-containing medium. The size measured for the mPEG-b-PLGA nanoparticles incubated with HPF did not vary with time, while an average increase of 65% was detected for the PLGA-COOH nanoparticles. For the PLGA-COOH + sodium cholate nanoparticles, we measured an average size increase of 3300% in the first 35 min of incubation, and then we supposed progressive precipitation of the nanoparticles interacting with the protein, and the size of the residual suspended nanoparticles progressively increased up to 260% in the last 20 min of incubation. The precipitation of very large HPF-coated nanoparticles was also confirmed by the trend of the mean photon count, which showed a sharp decrease at the incubation time of 35 min. The average size of the nanoparticles incubated with HBG increased by 24% (PLGA-COOH), 16% (mPEG-b-PLGA), and 74% (PLGA-COOH + sodium cholate).

The optical density of the nanoparticle/protein mixtures, measured by UV-Vis spectroscopy, was also in line with the results obtained with the other experimental techniques. The optical density of the samples incubated with the HSA did not significantly vary in the PLGA-COOH and mPEG-b-PLGA nanoparticle samples, while a small increase was detected for the PLGA-COOH + sodium cholate nanoparticles (10% after 60 min). For all the samples incubated with the HPF, an OD increase of 25–32% was measured after 30 min. This was also the case for the mPEG-b-PLGA nanoparticles. After 60 min, the percent variations in the OD were 6% (PLGA-COOH), 22% (mPEG-b-PLGA), and −6% (PLGA-COOH + sodium cholate). The negative value measured for PLGA-COOH + sodium cholate can be attributed to the visible flocculation of the HPF interacting with the nanoparticles. However, the decrease in the ΔOD measured for the PLGA-COOH nanoparticles at 30 min (+25%) and 60 min (+6%) probably indicated potential precipitation also for this sample.

## 5. Conclusions

QCM-D measurements can provide accurate information on the interaction between blood proteins and polymer nanoparticles. Our QCM-D data were compared to those obtained with more standard techniques to evaluate nanoparticle/protein interactions, and all the data are in line, confirming the goodness of QCM-D for this study. The obtained data were in line with the literature, with the surface chemistry governing the nanoparticle/protein interactions. The properties of hydrophobic surfaces, such as that of PLGA-COOH nanoparticles, can be improved by PEGylation, while the use of a surfactant, such as sodium cholate, can worsen the compatibility with blood proteins.

These experiments provide insight into blood nanoparticle/protein interactions, giving broad indications of potential in vivo behavior. Overall, this research contributes to a better understanding of protein corona analysis and its role in the development of safe and effective nanoparticles for biomedical applications.

In conclusion, the strategy reported can be considered a preliminary analysis of nanoparticle hemocompatibility.

## Data Availability

The data will be shortly available in a public database.
